# Genetic diversity of *Blastocystis* in non-primate animals

**DOI:** 10.1017/S0031182017002347

**Published:** 2018-01-17

**Authors:** Emma L. Betts, Eleni Gentekaki, Adele Thomasz, Vicki Breakell, Angus I. Carpenter, Anastasios D. Tsaousis

**Affiliations:** 1 Laboratory of Molecular and Evolutionary Parasitology, RAPID group, School of Biosciences, University of Kent, Canterbury, Kent, UK; 2 School of Science and Human Gut Microbiome for Health Research Unit, Mae Fah Luang University, Chiang Rai, Thailand; 3 Wildwood Trust, Herne Common, Herne Bay, Kent, UK

**Keywords:** *Blastocystis*, genetic diversity, phylogeny, prevalence, subtype

## Abstract

*Blastocystis* is an anaerobic protist, commonly inhabiting the intestinal tract of both humans and other animals. *Blastocystis* is extremely diverse comprising 17 genetically distinct subtypes in mammals and birds. Pathogenicity of this enteric microbe is currently disputed and knowledge regarding its distribution, diversity and zoonotic potential is fragmentary. Most research has focused on *Blastocystis* from primates, while sampling from other animals remains limited. Herein, we investigated the prevalence and distribution of *Blastocystis* in animals held within a conservation park in South East England. A total of 118 samples were collected from 27 vertebrate species. The barcoding region of the small-subunit ribosomal RNA was used for molecular identification and subtyping. Forty one per cent of the species were sequence positive for *Blastocystis* indicating a high prevalence and wide distribution among the animals in the park. Six subtypes were identified, one of which is potentially novel. Moreover, the majority of animals were asymptomatic carriers, suggesting that *Blastocystis* is not pathogenic in animals. This study provides a thorough investigation of *Blastocystis* prevalence within a wildlife park in the UK and can be used as a platform for further investigations on the distribution of other eukaryotic gut microbes.

## Introduction

*Blastocystis* is a microbial eukaryote that inhabits the gastrointestinal tract of a variety of animals including humans, other primates, amphibians, reptiles and even insects (Abe, 2004; Stensvold *et al*. 2009; Parkar *et al*. 2010; Roberts *et al*. 2013; Yoshikawa *et al*. 2016). After fungi, *Blastocystis* is one of the most prevalent microbial eukaryotes in metazoans (Scanlan *et al*. 2014).

Until recently, *Blastocystis* was overlooked due to its small size and lack of distinct morphological features. Nonetheless, the advent of molecular methods has revealed an astounding genetic heterogeneity of *Blastocystis*. To date, 17 genetically diverse lineages have been reported in mammals and birds (subtypes; ST), based on the differences of the small subunit ribosomal RNA (SSU rRNA) (Stensvold and Clark, 2016). *Blastocystis* has wide host range, with the same subtype found in several animal genera. Emerging data, however, suggests that host specificity should be assessed based on lower than genus level taxonomy (Alfellani *et al*. 2013*c*). Of the 17 STs, only the first nine (ST1–ST9) and recently, ST12 have been found in humans (Ramirez *et al*. 2016; Stensvold and Clark, 2016). *Blastocystis* has been reported in wild animals, pets and domesticated animals and those that are housed in zoos (Parkar *et al*. 2010; Ruaux and Stang, 2014; Schar *et al*. 2014; Wang *et al*. 2014; Amenu *et al*. 2015; Figueroa, 2015; Puebla *et al*. 2017). Nonetheless, the comprehensive range of non-primate hosts of the various STs remains unclear, since only a limited number of studies focus on screening such animals (Abe *et al*. 2002; Lim *et al*. 2008; Perez Cordon *et al*. 2008; Parkar *et al*. 2010; Roberts *et al*. 2013).

The presence in various animals of *Blastocystis* isolates that belong to the same STs as those in humans has led to the speculation that the organism has zoonotic potential (Rajah Salim *et al.* 1999; Parkar *et al*. 2010; Ramirez *et al*. 2016). Nonetheless, this scenario has come under scrutiny in recent years, since cases where the direction of transmission has been established conclusively are absent. Moreover, most molecular investigations of *Blastocystis* isolates from domesticated animals and their keepers have not revealed any shared subtypes, though there are notable exceptions (Alfellani *et al*. 2013*b*; Wang *et al*. 2014). Due to this controversy, there is an urgent need for further investigations on the distribution of *Blastocystis* in animals in captivity, since prevalence data and molecular characterization of *Blastocystis* in such animals remain sparse.

Herein, we examine *Blastocystis* isolates from Wildwood Trust, a wildlife park in East Kent, UK. The park's collection consists mostly of UK native and previously native wildlife, meaning that the chance of the identified isolates being local is very high. The aim of this study was to investigate the prevalence, distribution, genetic diversity and host range of *Blastocysti*s STs in animals at Wildwood Trust.

## Materials and methods

### Study site – source of specimens

A total of 118 faecal samples were collected from 27 different host species (Table 1) located at Wildwood Trust (Herne Bay, Kent, UK). Sampling covered a range of mammalian species, four bird species and one reptile species (Table 1).

**Table 1.** Animal samples collected from study hosts

Host	Scientific name	Location	Sample number	PCR positive (clone number)
Carnivora				
Badger	*Meles meles*	Wildwood	2	–
European Brown Bear	*Ursus arctos arctos*	Wildwood	2	–
Lynx	*Lynx lynx*	Wildwood	3	–
Otter	*Lutra lutra*	Wildwood	7	–
Pine Marten	*Martes martes*	Wildwood	2	1 [1]
Polecat	*Mustela putorius*	Wildwood	1	–
Red Fox	*Vulpes vulpes*	Wildwood	3	–
Scottish Wild Cat	*Felis silvestris*	Wildwood	11	–
Stoat	*Mustela ermine*	Wildwood	3	–
Anseriformes				
Barnacle Goose	*Branta leucopsis*	Wildwood	1	–
Pink Footed Goose	*Anser brachyrhynchus*	Wildwood	1	–
Artiodactyla				
Muntjac Deer	*Muntiacus reevesi*	Wildwood	1	1 [1]
European Bison	*Bison bonasus*	Wildwood	3	3 [11]
Eurasian Elk	*Alces alces*	Wildwood	2	1 [4]
Pygmy Goat	*Capra aegagrus hircus*	Wildwood	2	2 [3]
Red Deer	*Cervus elaphus*	Wildwood	1	1 [8]
Reindeer	*Rangifer tarandus*	Wildwood	1	–
Soay Sheep	*Ovis aries*	Wildwood	1	1 [1]
Wild Boar	*Sus scrofa*	Wildwood	2	1 [1]
Squamata				
Four-lined Snake	*Elaphe quatuorlineata*	Wildwood	1	–
Eulipotyphla				
Hedgehog	*Erinaceus europaeus*	Wildwood	1	–
Water Shrew	*Neomys fodiens*	Wildwood	6	–
Passeriformes				
Raven	*Corvus corax*	Wildwood	3	–
Red Billed Chough	*Pyrrhocorax pyrrhocorax*	Wildwood	1	–
Rodentia				
Red Squirrel	*Sciurus vulgaris*	Wildwood	3	2 [1]
Water Vole	*Arvicola amphibious*	Wildwood	3	2 [12]
Water Vole	*Arvicola amphibious*	Bulphan	(5) 30^a^	5 [17]
Water Vole	*Arvicola amphibious*	Tilbury	(3) 18^a^	3 [9]
Diprotodontia Wallaby	*Macropus rufogriseus*	Wildwood	3	2 [2]

a High sample number due to repetitive sampling from a small population. Numbers in parentheses denote number of water vole individuals.

### Sample collection and storage

A licensed veterinarian visits the park on a monthly basis to monitor the animals’ health, during the week of sampling no animals exhibit diarrhoea. Faecal samples were collected between the months of July 2016 to January 2017. Wildwood Trust staff collected samples under the guidance of laboratory members; A minimum of one sample was collected per animal species, where possible (Table 1). In the cases where multiple animals of the same species were enclosed together, several samples were collected.

Once collected, samples were placed in sealed, sterile falcon tubes and stored at 4 °C until DNA extraction. The faecal samples were subdivided shortly after collection to be used for microscopy, culturing and DNA extraction. Heat-fixed slides were made from all samples collected within an hour of collection.

### Culturing

Within half an hour of sampling, a small amount of faecal sample from randomly selected animals were separately inoculated in sterile falcon tubes containing the following media: two tubes containing modified LYSGM [16·07 mm potassium phosphate dibasic, 2·94 mm potassium phosphate monobasic, 128·34 mm sodium chloride, 2·5 g L^−1^ yeast extract, 0·5 g L^−1^ liver extract, 5% adult bovine (Sigma)/horse serum (Gibco); modified TYSGM-9, without mucin (Diamond, 1982), http://entamoeba.lshtm.ac.uk/xenic.htm], two tubes of TYM (22·2 g L^−1^ trypticase peptone, 11·1 g L^−1^ yeast extract, 16·23 mm maltose, 9·17 mm L-cysteine, 1·26 mm L-ascorbic acid, 5·1 mm potassium phosphate dibasic, 6·53 mm potassium phosphate monobasic) (Diamond, 1957, 1983) enriched with 5% fetal bovine serum (FBS; Sigma) and 2 tubes with 0·5% Liver Digest (LD) medium (0·5 g L^−1^ Oxoid liver extract). One tube of each medium type was incubated at 35 °C and the rest were left at room temperature. Samples were examined every 3 days under light microscope with neutral red staining (see below). Cultures positive for *Blastocystis,* were subcultured every 10 days.

### Staining and microscopy

For the identification of live cells within cultures, a neutral red staining technique was employed (DeRenzis and Schechtman, 1973). Ninety-four *μ*L of re-suspended cultured samples were mixed with equal volumes of freshly prepared 0·04% neutral red staining (Sigma, N2889) in 0·5 mL tubes and incubated for 10 min at the temperature in which samples were cultured. The samples were then centrifuged at 5000 ***g*** for 30 s. The supernatant was removed and the pellet was re-suspended in 20 *µ*L of 1 × PBS (pH 7·2) by vortexing. Ten *μ*L of the mixture was placed on a glass slide under a 22-mm square coverslip and individual cells were observed under 200× and 400× magnification.

### DNA extraction, PCR, cloning and sequencing

DNA from feces and cultures were extracted using the Microbiome DNA Purification Kit Purelink (Fisher, UK), following the manufacturer's specifications and protocols. The extracted DNA was stored at −20 °C for long-term usage. To amplify the fragment of interest, polymerase chain reaction (PCR) was carried out using the extracted DNA. DNA extracted from an axenic *Blastocystis* NandII culture was used as positive control in every PCR application. The conditions of amplification were as follows: 2 *µ*L of the extracted DNA was used for amplification of a *Blastocystis* sp SSUrRNA product. 10 *µ*L 5×  buffer (Promega), 1 mm MgCl_2_, 0·4 *µ*m forward primer, 0·4 *µ*m reverse primer, 0·2 mm dNTPs (Promega), 0·25 *µ*L Taq polymerase, 30·75 *µ*L HPLC grade water 2 *µ*L DNA. The fragment was amplified in a total of 50 *µ*L reaction, according to the standard conditions of for HiFI Taq polymerase (Promega). The broad specificity primers RD3 5′-GGGATCCTGATCCTTCCGCAGGTTCACCTAC-3′ and RD5 5′-GGAAGCTTATCTGGTTGATCCTGCCAGTA-3′ (Clark, 1997) were used for the first PCR. Cycling conditions were as follows: 95 °C 5 min, 35 cycles of denaturation at 95 °C for 30 s, annealing 55 °C for 30 s, extension at 72 °C for 1 min 40 s and final extension at 72 °C for 5 min.

A second nested PCR was performed using the forward RD5F 5′-ATCTGGTTGATCCTGCCAGT-3′ and reverse BhRDr 5′-GAGCTTTTTAACTGCAACAACG-3′ (Scicluna *et al*. 2006) primers giving a fragment at approximately 650 bp. This fragment is considered the barcoding region for *Blastocystis* identification. Concentration of reagents in each reaction and PCR conditions were the same as above. One *μ*L from the PCR mentioned above was used as template.

Positive PCR reactions from the nested-PCR were gel-extracted using the Thermo Scientific GeneJET Gel Extraction Kit (following manufacturer's instructions) and subsequently cloned in the pGEM-T vector (Promega) using the manufacturer's protocol. Five to ten colonies from each transformation were selected for sub culturing and plasmid purification using the GeneJET Plasmid Miniprep Kit. Positive plasmids were screened by digestion with EcoRI restriction enzyme, to confirm presence of the fragment of interest. Positive plasmids were bidirectionally sequenced using T7 and SP6 universal primers by Eurofins, UK.

### Genetic distance and phylogenetic analysis

The obtained sequences were trimmed to eliminate vector fragments and forward and reverse sequences of each sample were joined using Sequencher. Blast searches against GenBank using the sequences obtained were performed to exclude bacterial contamination. A dataset including all new sequences identified as *Blastocystis* along with sequences spanning the breadth of diversity of *Blastocystis* subtypes was build and aligned using MAFFT v.7 (Katoh and Toh, 2010). The alignment was further improved by visual check where necessary. Genetic distance was calculated using the Kimura2 parameter criterion. Gaps were considered as complete deletions. For this calculation, only the barcoding region of *Blastocystis* was used.

For the phylogenetic analysis, four additional outgroup taxa were included to the alignment and the entire sequence of SSUrRNA was used. The alignment contained a total of 90 taxa. Several sequences were represented only by their barcoding region in which case, the missing part of the sequence was considered as missing data. Following alignment with MAFFT, ambiguous positions were removed using trimAL (Capella-Gutierrez *et al*. 2009). After trimming the alignment contained 1163 sites. Phylogenetic trees were constructed by using maximum likelihood and Bayesian inference methods. Maximum likelihood trees were computed using the RAxML software (Stamatakis, 2006). For each dataset bootstrap support was evaluated from 1000 bootstrap replicates. Bayesian inference tree was computed using MrBayes (Ronquist and Huelsenbeck, 2003). Posterior probabilities were computed by running four chains with sampling occurring every 100th generation, whilst 25% of trees were discarded as burn-in. Trees were run for 1 500 000 generations at which point all parameters converged at 0·01.

## Results

### Culturing

*Blastocystis* grew in the tubes containing LYSGM and TYM + FBS at both 35 °C and room temperature. There was no *Blastocystis* growth in the 0·5% LD medium.

### Screening of faecal samples

A total of 118 samples from 27 species were examined of which 71 clones were sequence positive belonging to 11 species (41%) (Table 1). Nonetheless, there was a notable difference in the presence of *Blastocystis* across hosts. With the exception of a single case, all sequence positive samples came from non-carnivorous animals. This was despite repeated sampling and sequencing attempts (Table 1). Specifically, 7/8 (87·5%) of artiodactyls, 2/2 (100%) of rodents and 1/9 (11%) of carnivores were sequence positive for *Blastocystis*. No sequence positive samples were found in birds, snakes and insectivores (Table 1).

### Subtype identification and distribution in various hosts

**Fig. 1.**
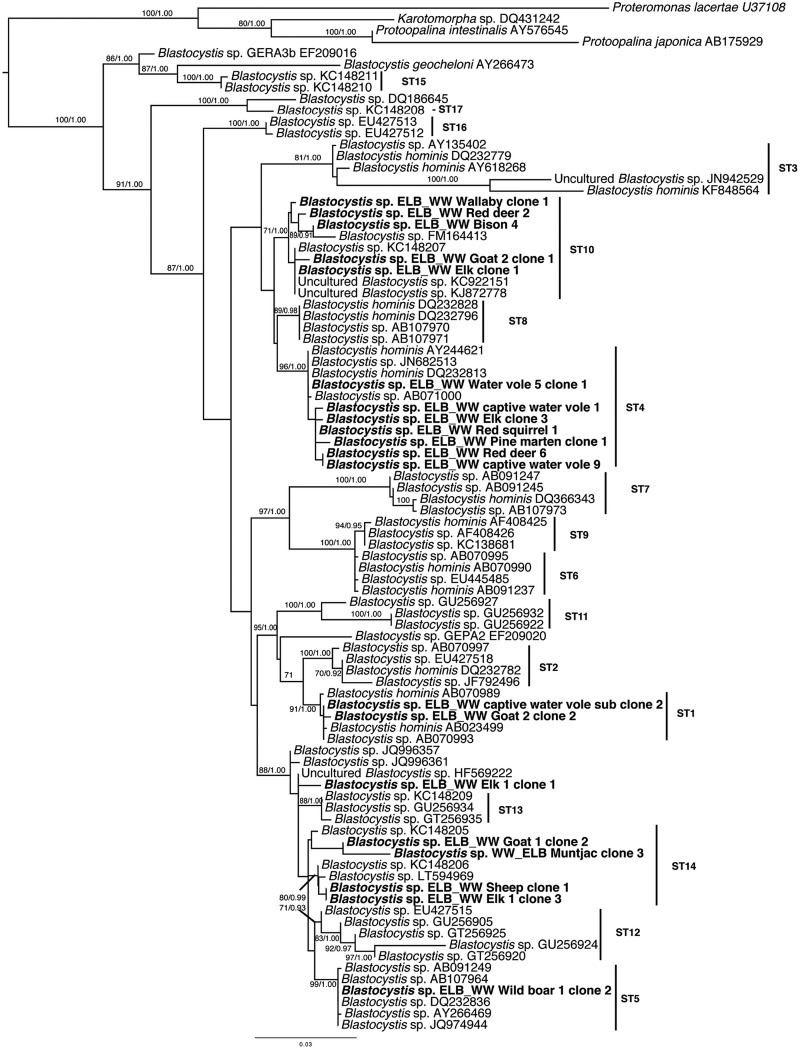
Maximum likelihood phylogenetic tree inferred from 90 SSUrRNA sequences and 1163 sites. Newly generated sequences are shown in bold. Numerical values on the branches indicate bootstrap percentages and posterior probabilities in this order. Only bootstrap support values greater than 70 are shown. The accession numbers of all newly generated sequences are presented in online Supplementary Table S1.

Among the 71 *Blastocystis*-positive samples, six STs were detected (Table 2, Fig. 1): ST1, ST4, ST5, ST10, ST14 and a potentially new subtype. Subtypes 4 and 10 colonized the most species (seven and six respectively) followed by ST14 (three), ST1 (two), ST5 (one) and a novel subtype (one). We provide the first molecular data and subtyipng of *Blastocystis* from elk, water voles, pine martens and red squirrels. The Eurasian elk (artiodactyl) were the hosts harbouring the widest range of subtypes, followed by pygmy goat (artiodactyl) and water vole (rodent). Most notably, four subtypes were found in the elk (ST4, ST10, ST14, novel), while goat and water vole harboured three (ST1, ST10 and ST14 in goat and ST1, ST4 and ST10 in water vole). The hosting of multiple subtypes within elk is of no doubt, as there is just a single elk in the park. The same cannot be verified for the goats and voles as the park houses several of them. Nonetheless, only two faecal samples were collected, which means that there are at least two subtypes present in a single goat. The three subtypes in water vole were identified only in the captive population of which three were sampled. The presence of all subtypes can be confirmed here, due to cloning being used rather than PCR purification of a single product.

**Table 2.** Subtype results from sequencing positive samples

					*Blastocystis* ST					
Host	Name	Location	PCR positive samples	Sequence positive clones	ST1	ST4	ST5	ST10	ST14	ST?
European Bison	*Bison bonasus*	Wildwood	3	11	–	–	–	11/11	–	–
Eurasian Elk	*Alces alces*	Wildwood	1	4	–	1/4	–	1/4	1/4	1/4
Muntjac Deer	*Muntiacus reevesi*	Wildwood	1	1	–	–	–	–	1/1	–
Pine Marten	*Martes martes*	Wildwood	1	1	–	1/1	–	–	–	–
Pygmy Goat	*Capra aegagrus hircus*	Wildwood	2	3	1/3	–	–	1/3	1/3	–
Red Deer	*Cervus elaphus*	Wildwood	1	8	–	2/8	–	6/8	–	–
Red Squirrel	*Sciurus vulgaris*	Wildwood	2	1	–	1/1	–	–	–	–
Soay Sheep	*Ovis aries*	Wildwood	1	1	–	–	–	–	1/1	–
Wallaby	*Macropus rufogriseus*	Wildwood	2	2	–	–	–	2/2	–	–
Water Vole	*Arvicola amphibius*	Wildwood	2	12	1/12	10/12	–	1/12	–	–
Water Vole PP	*Arvicola amphibius*	Bulphan	5	17	–	17/17	–	–	–	–
Water Vole TB	*Arvicola amphibius*	Tilbury	3	9	–	9/9	–	–	–	–
Wild Boar	*Sus scrofa*	Wildwood	1	1	–	–	1/1	–	–	–

Several samples were collected from two rodent species; the red squirrel and water vole. Subtype 4 was commonly detected in both species, while the range of subtypes previously reported within rodents can be expanded to include ST1. Several colonies were also screened from wallabies, diprotodontid marsupials. All samples from wallabies harboured ST10, which had not been reported previously from these marsupials.

### Phylogenetic analysis

Though 71 clones were sequenced, only 20 of them were used in the phylogenetic analyses. In the cases where clusters contained identical clones, only a few representative sequences were kept. In total, the new sequences were subtyped as follows: ST4 (*n* = 41); ST10 (*n* = 22); ST14 (*n* = 4); ST1 (*n* = 2); ST5 (*n* = 1) novel ST (*n* = 1). All *Blastocystis* sequences formed a strongly supported cluster (100BS/1·00BI). Most newly sequenced isolates grouped within clades formed by previously published subtypes (Fig. 1). The most basal sequences belonged to *Blastocystis* isolates from reptiles and cockroaches along with those from ST15, ST16 and ST17 in agreement with previous studies (Alfellani *et al*. 2013*c*; Yoshikawa *et al*. 2016). Subtype 3 sequences grouped together and sister to a clustered formed by ST10, ST8 and ST4. Subtypes 7, 9 and 6 clustered together, while ST11, ST2 and ST1 formed a separate clade. Subtypes 12 and 5 also grouped together. Subtypes 13 and 14 were not well resolved even when a subtree was constructed (data not shown). The ELB_WW Elk 1 clone 1 did not fall within any of the 17 STs and its position remains unresolved.

## Discussion

Approximately 61 animals from 27 species were examined. Forty one per cent of all animals were sequence positive for *Blastocystis*. In select cases, we attempted to establish cultures of *Blastocystis*. The organism has been previously cultured in a wide range of media including egg slant medium with Locke's solution, Iscove's modified Dulbecco's medium, Robinson's medium and Jones’ medium (Clark and Diamond, 2002; Tan, 2008). The latter is a widely used formulation ideal for short term culturing of multiple subtypes (i.e. a few days). *Blastocystis* isolates originating from endothermic hosts are customarily cultured at 35 °C. Reported here was cultivation of *Blastocystis* from a water vole (*Arvicola amphibius*) in TYM medium enriched with FBS. The culture had been maintained in the laboratory for at least 11 months. Although the origin of the isolate is an endothermic animal, it grew over abundantly at room temperature. This indicates that some isolates of *Blastocystis* can grow at lower temperatures given certain types of media. Whether all isolates of *Blastocystis* or only some can grow in TYM + FBS at room temperature needs further study.

Most of the animals that we examined harboured a single subtype of *Blastocystis*. Nonetheless, some animals carried more than one subtype. Mixed colonization was confirmed, because we employed cloning and screened multiple colonies from each sample, while previous studies only used direct sequencing from PCR products (Stensvold *et al.* 2012; Roberts *et al.* 2013; Alfellani *et al.* 2013*b*, *c*; Stensvold, 2013). Using this strategy, it was found that elk (*Alces alces*) harboured four subtypes, with this being the first time *Blastocystis* has been reported in this mammal. In cases where multiple subtypes are found within a single host, it is important to exclude contamination from other sources. The park has a single elk, which is housed in an isolated enclosure on its own. Moreover, the faecal sample was collected at the moment of defecation precluding contamination from small, non-resident animals. More than one subtype was also detected in pygmy goats (ST = 3), red deer (ST = 2) and water voles (ST = 3). Unlike in the case of the elk, we cannot definitively conclude that the detected subtypes in goats originated from a single individual *per se*, since enclosures housed multiple animals of the same species. While colonization with multiple subtypes is rare in humans, not much information is available for other animal species (Meloni *et al*. 2012). In light of our findings, it is tempting to speculate that the microbiota of at least some animals includes *Blastocystis* subtypes. Sampling from more animals and use of methodologies similar to ours will shed further light as to whether presence of multiple subtypes is the norm within these and other animals.

Water voles also constitute an interesting case. There are two, temporary populations of water vole being held within the park, together with permanent residents. These two groupings of water vole are temporarily brought in to captivity as part of a licensed, development mitigation programme and are subsequently to be introduced back into their natural environment locations; two separate sites in Essex, UK. This study can report that ‘wild’ water vole harboured ST4 only, whereas those in permanent captivity also harboured ST1. Wild water voles were sampled multiple times, while captive ones provided only a limited number of samples. Despite considerable effort (PCR, cloning and screening of clones) we were unable to detect ST1 in wild water voles. It is tempting to speculate that the ‘captive’ water vole acquired ST1 after their introduction in the park and that this is one of the many microbiota-related alterations associated with life in captivity (Waite and Taylor, 2014; Kohl *et al*. 2017). However, since captive voles originated from two additional locations other than Essex, this hypothesis needs further testing involving surveys of all populations of origin.

ST10 and ST4 were the most widely distributed subtypes, each isolated from five species. As previously described, artiodactyls carried mostly ST10 (Alfellani *et al*. 2013*c*). It has been speculated that rodents are reservoirs of ST4 for human infection, though not all rodent species carry this specific ST (Alfellani *et al*. 2013*b*). Subtypes 3 and 17 were also found in rodents in previous investigations (Stensvold *et al*. 2009; Alfellani *et al*. 2013*a*). Herein, this study detected ST4 in all *Blastocystis* positive samples of rodents. Nonetheless, other subtypes were also found in the screened rodents: ST10 in red squirrels and ST1, ST5 and ST10 in water voles. Therefore, the study has been able to expand the number of subtypes recorded in rodents by identifying ST1 and ST10. It was also possible to expand the range of subtypes identified in goats to include ST14, along with the previously identified ST10, ST1, ST3, ST6 and ST7 (Alfellani *et al*. 2013*b*). The study also detected ST14 in four hosts, all of which belong to the artiodactyls.

To determine the monophyly and relationships among STs, phylogenetic analyses were performed. Traditionally, sampling of *Blastocystis* had focused on primates, especially humans. As a result, STs that were present in non-primates were reported infrequently and the clades of these STs remained sparsely populated. For instance, the resolution of the ST13 and ST14 has been problematic. Previously, Alfellani *et al*. (2013*c*) speculated that ST14 should be split into two subtypes, but refrained from doing so pending further sampling. The current study has shown that, when our isolates were added to the tree, ST14 splits into two distinct clades, with our samples populating both of these clades, hence, supporting the idea that it should be considered as two STs. Moreover, one isolate from elk grouped independently of all other STs, suggesting that this might be a novel ST. Genetic divergence analysis of the barcode region indicated that the genetic distance between our isolate and all other STs is over 5%, with the exception of ST13, with which it differed by 4·4%. The recommended threshold to define a new sequence is 5% divergence (Clark *et al*. 2013). Nonetheless, the full sequence and further samples are needed to confirm this finding since this is an individual partial sequence.

In summary, we present here a comprehensive study of *Blastocystis* prevalence focusing exclusively on non-primate animals in a zoo setting in the UK. Presented here has been the presence of six subtypes, with one potentially being novel. Through the use of cloning, it has been possible to conclusively record the presence of multiple STs within an individual animal. The sequences generated from this study have populated STs that were considered rare and for which not many sequences exist. Collectively, these highlight the need for sampling from a wide range of hosts.

## Supplementary material

For supplementary material accompanying this paper visit http://dx.doi.org/10.1017/S0031182017002347. click here to view supplementary material
