# Supplementary material for: Genetic diversity of Blastocystis in non-primate animals
Source: Parasitology. 2018 Jan 17;145(9):1228–34. doi: 10.1017/S0031182017002347 (PMC5912512; doi:10.1017/S0031182017002347)
Supplement: Supplementary file 1 [file S0031182017002347sup001.docx]

**Table S1:** Corresponding accession numbers to the sequences shown in the phylogenetic tree (Figure 1).

| **Animal** | **Accession number** |
| --- | --- |
| ELB_WW_Water vole 1_ clone 1 | MF186640 |
| ELB_WW_Water vole 3_clone 3 | MF186641 |
| ELB_WW_Water vole 2_clone 3 | MF186642 |
| ELB_WW_Water vole 2_clone 2 | MF186643 |
| ELB_WW_Water vole 1_ clone 2 | MF186644 |
| ELB_WW_Captive Water vole 3_Subculture_clone 4 | MF186645 |
| ELB_WW_Captive Water vole 1_Subculture_clone 3 | MF186646 |
| ELB_WW_Captive Water vole 1_Subculture_clone 2 | MF186647 |
| ELB_WW_Water vole 34_clone 3 | MF186648 |
| ELB_WW_Water vole 34_clone 1 | MF186649 |
| ELB_WW_Water vole 34_clone 2 | MF186650 |
| ELB_WW_Water vole 32_clone 3 | MF186651 |
| ELB_WW_Water vole 32_clone 2 | MF186652 |
| ELB_WW_Water vole 32_clone 1 | MF186653 |
| ELB_WW_Water vole 30_clone 3 | MF186654 |
| ELB_WW_Water vole 30_clone 2 | MF186655 |
| ELB_WW_Water vole 30_clone 1 | MF186656 |
| ELB_WW_Water vole 5_clone 2 | MF186657 |
| ELB_WW_Water vole 5_clone 3 | MF186658 |
| ELB_WW_Water vole 5_clone 1 | MF186659 |
| ELB_WW_Water vole 5_clone 5 | MF186660 |
| ELB_WW_Water vole 4_clone 3 | MF186661 |
| ELB_WW_Water vole 4_clone 3 | MF186662 |
| ELB_WW_Water vole 4_clone 1 | MF186663 |
| ELB_WW_Elk 1_clone 3 | MF186664 |
| ELB_WW_Elk 1_clone 1 | MF186665 |
| ELB_WW_Bison_1 | MF186666 |
| ELB_WW_Red squirrel_1 | MF186667 |
| ELB_WW_Red deer_1 | MF186668 |
| ELB_WW_Red deer_2 | MF186669 |
| ELB_WW_Captive Water vole_1 | MF186670 |
| ELB_WW_Red deer_3 | MF186671 |
| ELB_WW_Bison_2 | MF186672 |
| ELB_WW_Captive Water vole_2 | MF186673 |
| ELB_WW_Captive Water vole_3 | MF186674 |
| ELB_WW_Bison 2_1 | MF186675 |
| ELB_WW_Bison_3 | MF186676 |
| ELB_WW_Red deer_4 | MF186677 |
| ELB_WW_Bison_4 | MF186678 |
| ELB_WW_Red deer_5 | MF186679 |
| ELB_WW_Bison 2_2 | MF186680 |
| ELB_WW_Bison_5 | MF186681 |
| ELB_WW_Bison_6 | MF186682 |
| ELB_WW_Red deer_6 | MF186683 |
| ELB_WW_Captive Water vole_4 | MF186684 |
| ELB_WW_Red deer_7 | MF186685 |
| ELB_WW_Bison_7 | MF186686 |
| ELB_WW_Captive Water vole_5 | MF186687 |
| ELB_WW_Bison_8 | MF186688 |
| ELB_WW_Red deer_8 | MF186689 |
| ELB_WW_Captive Water vole_6 | MF186690 |
| ELB_WW_Captive Water vole_7 | MF186691 |
| ELB_WW_Captive Water vole_8 | MF186692 |
| ELB_WW_Captive Water vole_9 | MF186693 |
| ELB_WW_Wallaby_1 | MF186694 |
| ELB_WW_Bison_SP6_clone 1 | MF186695 |
| ELB_WW_Elk_clone 1 | MF186696 |
| ELB_WW_Elk_clone 3 | MF186697 |
| ELB_WW_Goat 2_clone 1 | MF186698 |
| ELB_WW_Goat 2_clone 2 | MF186699 |
| ELB_WW_Muntjac_clone 3 | MF186700 |
| ELB_WW_Pine marten_clone 1 | MF186701 |
| ELB_WW_Water vole 1_clone 3 | MF186702 |
| ELB_WW_Water vole 2_clone 1 | MF186703 |
| ELB_WW_Water vole 3_clone 2 | MF186704 |
| ELB_WW_Water vole 3_clone 3 | MF186705 |
| ELB_WW_Water vole 5_clone 3 | MF186706 |
| ELB_WW_Sheep_clone 1 | MF186707 |
| ELB_WW_Wallaby_clone 1 | MF186708 |
| ELB_WW_Wild boar_clone 1 | MF186709 |
| ELB_WW_Goat 1_clone 1 | MG592389 |
